# Acceptability and Use of Digital Health and Artificial Intelligence–Enabled Chatbots for Sexual and Reproductive Health Among Lesbian, Bisexual, and Queer Women of Color in the United States: Cross-Sectional Survey Study

**DOI:** 10.2196/84393

**Published:** 2025-12-29

**Authors:** Megan Threats, Morgan Gray

**Affiliations:** 1 School of Information, University of Michigan, 2200 Hayward Street, Ann Arbor, MI, 48109, United States, 1 734-647-0826

**Keywords:** digital health, artificial intelligence, ethnic and racial minorities, health equity, sexual health, reproductive health, sexual and gender minorities, mHealth, women, cervical cancer

## Abstract

**Background:**

Cisgender lesbian, bisexual, and queer (LBQ+) women of color experience barriers to accessing sexual and reproductive health (SRH) services in the United States. Barriers, including limited provider access and poor patient-provider communication, contribute to SRH service underutilization and poorer outcomes among these women than their heterosexual counterparts. Digital health modalities, including telemedicine, mobile health, and chatbots enabled by artificial intelligence (AI), offer potential to expand access to SRH information and services among these women.

**Objective:**

This study investigated the influencing factors, acceptability, and concerns regarding the use of digital health modalities (video calls, SMS text messaging, and mobile apps) and AI-enabled chatbots to support SRH information and service access among LBQ+ women of color in the United States. It also assessed their awareness and knowledge of human papillomavirus (HPV) and cervical cancer prevention, and attitudes toward HIV prevention medication.

**Methods:**

A self-administered online survey was conducted from November 2020 to March 2021 with 285 LBQ+ women of color (aged ≥18 years) residing in the United States. The 88-item survey assessed digital health use, SRH knowledge and awareness, and acceptability of and concerns about digital health use for SRH information and services. Data were analyzed using descriptive statistics, Fisher exact tests, multivariable logistic regression, and thematic analysis.

**Results:**

Most respondents (233/285, 81.8%) were comfortable using video calls to communicate with health care providers for SRH support. Respondents with a bachelor’s degree or higher (95% CI 0.00‐0.24), with health insurance (95% CI 56.1‐1025.7), and without a usual place of care (95% CI 0.07‐0.43) were significantly (*P*<.001) more likely to agree with using video calls. Respondents with a bachelor’s degree or higher (95% CI 0.23‐0.74), aged <45 years (95% CI 0.07‐0.25), and with health insurance (95% CI 3.23‐12.45) were significantly (*P*<.001) more likely to agree with using mobile apps. Respondents aged ≥45 years (95% CI 0.14‐0.53), without health insurance (95% CI 0.01‐0.06), and with an income of <US $49,000 (95% CI 1.32‐3.93) were significantly (*P*<.001) more likely to agree with the use of SMS text messaging. There was high acceptance of using chatbots for self-assessing sexually transmitted infection risk (229/285, 80.3%) but lower acceptance for self-assessing cervical cancer risk (136/285, 47.7%). Key concerns included data privacy and confidentiality, lack of affective communication, and technology connectivity and digital literacy issues. Respondents also demonstrated low knowledge of HPV and cervical cancer prevention.

**Conclusions:**

Digital health was highly acceptable for supporting access to SRH information and services among LBQ+ women of color. Culturally tailored digital tools and interventions could improve awareness, knowledge, and attitudes toward SRH services. Addressing various digital literacy levels, data privacy concerns, and technology access and communication issues when developing digital health solutions may advance SRH equity among LBQ+ women of color.

## Introduction

There is limited research examining the sexual and reproductive health (SRH) experiences of cisgender, lesbian, bisexual, and queer (LBQ+) women of color in the United States. Historically, research about the SRH experiences of sexual minority adults has focused on HIV and AIDS among sexual minority men, with a limited focus on LBQ+ women of color and diseases such as cancer [1-3]. Similarly, LBQ+ women of color are underrepresented in SRH research among women of color, which has primarily focused on heterosexual women [3,4]. Prevailing and misguided stereotypes rooted in heterosexism assume that LBQ+ women are not likely to acquire sexually transmitted infections (STIs) or have unintended pregnancies [3,5]. However, emerging research suggests that the limited focus on the SRH of LBQ+ women of color has given rise to disparities in their SRH outcomes.

SRH inequities persist in the United States, disproportionately affecting Black populations and other racial/ethnic minority populations [6,7]. Emerging research suggests that LBQ+ women of color experience racial/ethnic and sexual orientation disparities in access to human papillomavirus (HPV) vaccination and screening, cervical cancer screening, HIV and STI testing, breast cancer screening, and contraception [8-12]. In comparison to their White heterosexual counterparts, LBQ+ women of color are less likely to use SRH services due to barriers such as lack of health insurance, lack of a usual health care source, and discrimination in health care settings [13-16]. Due to these barriers, research indicates that LBQ+ women of color experience poorer health outcomes, including delayed care, late diagnosis of HIV and STIs, diagnosis of cancers at later stages, and higher incidence and mortality rates [17-22].

While there is limited research investigating the SRH experiences of LBQ+ women of color, existing research suggests that they report limited access to SRH information; patient-provider communication; and care that centers on their information needs, communication preferences, priorities, and cultural norms [23-25]. LBQ+ women of color report experiences of interpersonal discrimination in health care settings due to the racist, classist, weight-based, and heterosexist biases of health care providers [15,23,26]. Previous research has shown that LBQ+ women of color feel shamed, unheard, and dismissed during their interactions with health care providers, which has led to them delaying or forgoing the use of SRH services [24,27-29]. Improving access to and uptake of SRH services and information among LBQ+ women of color is critical for improving health care outcomes and reducing disparities.

Health literacy, defined as “the degree to which individuals have the ability to find, understand, and use information and services to inform health-related decisions and actions for themselves and others,” is critical for SRH [30,31]. Before adopting SRH services, individuals must be aware that these services exist and must possess the knowledge and skills necessary to make an informed decision about using them [32]. Poor knowledge and awareness of SRH services may contribute to lower use of SRH prevention services [33], increased risk of advanced disease at diagnosis [12,34], and greater susceptibility to misinformation due to incorrect beliefs about their risk for STIs, including HIV and HPV, and cervical cancer [35]. Identifying gaps in knowledge and awareness of SRH services among LBQ+ women of color, such as STI and cervical cancer prevention methods, may help optimize their SRH outcomes [36].

Digital health modalities, such as mobile apps and SMS text messaging, may help reduce disparities in access to and uptake of SRH services and information among LBQ+ women of color in the United States [37-39]. While empirical data on the use of digital health modalities among LBQ+ women of color are limited, existing studies have shown that LBQ+ women use mobile apps for seeking information [40], building social support [41], and seeking romantic partnerships [42]. Past digital health interventions using modalities, such as SMS text messaging [43,44] and mobile apps [45,46], increased the uptake of SRH services among heterosexual women of color and improved their knowledge of HIV and other STIs [47,48].

Chatbots enabled by artificial intelligence (AI) and telehealth video calls also hold promise for increasing access to SRH services and information [37,49,50]. LBQ+ women of color who experience geographical and logistical barriers in accessing SRH services face-to-face or who anticipate discrimination in health care settings and desire nonstigmatizing or nonjudgmental SRH information and referrals to inclusive care, may find these modalities useful [29,51]. Additionally, given that LBQ+ women of color have a higher likelihood of contracting HPV and being diagnosed with cervical cancer than heterosexual women, new approaches to improving care access are needed [52-56].

There is a growing body of research on digital health equity that seeks to improve our understanding of how digital health may improve health care access, use, and patient experiences, and reduce health disparities among underserved and historically marginalized populations [57-63]. Kim and Backonja [63] defined digital health equity as “a multilevel socioecological concept that results from fair and just opportunities for everyone to attain their highest level of health through access to technology-enabled health resources and services.” Research into the willingness of sexual minority adults to use digital health modalities, as well as their effectiveness for advancing health equity, has primarily focused on HIV prevention and treatment intervention efforts for cisgender sexual minority men [64,65]. Similarly, the majority of digital health equity research that is centered on supporting the health and well-being of women of color has focused on heterosexual women [46,66,67]. There is a need for digital health equity research that is more inclusive of the experiences and perspectives of LBQ+ women of color [67-70]. Addressing disparities in SRH among LBQ+ women of color using digital health requires filling this critical gap in research to avoid the development of biased, ineffective, or even harmful interventions [71-73].

Therefore, to address this gap, the study investigated the current use, influencing factors (ie, sociodemographic characteristics), acceptability, and concerns of digital health modalities (video calls, mobile apps, and SMS text messaging) and AI-enabled chatbots for supporting SRH information and service access. It also assessed the awareness and knowledge of HPV and cervical cancer prevention, and the attitudes and awareness regarding HIV prevention medication among LBQ+ women of color in the United States. We chose to focus on this area due to existing research indicating that LBQ+ women of color are at a heightened risk of acquiring HPV and being diagnosed with cervical cancer. Additionally, given the success of digital health modalities in enhancing SRH awareness and knowledge (ie, health literacy) and improving SRH outcomes for similar populations (eg, sexual minorities and racial/ethnic minorities), we assessed their utility and the demographic factors influencing their potential uptake and use [74]. To our knowledge, this is the first study to explore this phenomenon in this understudied population.

## Methods

### Recruitment and Sampling

The study included a convenience sample of women of color (aged ≥18 years) who identified as LBQ+ and resided in the United States. These women were required to complete a cross-sectional, self-administered online survey. The survey was launched in November 2020 and closed in March 2021. To be eligible, participants needed to identify as American Indian, Asian, Black/African American, Hispanic/Latina, Middle Eastern/North African, Native Hawaiian, or other Pacific Islander; be assigned female at birth; identify as an LBQ+ woman; be aged 18 years or older; and live in the United States or a US territory (eg, Puerto Rico). Respondents were recruited through social media posts (eg, Twitter, Instagram, and Facebook) and via an anonymous link distributed to community-based organizations and university study group listservs, which had members who identified as LBQ+ women of color. A snowball sampling technique was used to encourage respondents to share the anonymous survey link with members of their networks who fit the eligibility criteria for the study. The CHERRIES (Checklist for Reporting Results of Internet E-Surveys) checklist is provided in Checklist 1.

### Ethical Considerations

This study received approval for all research activities from the Rutgers University Institutional Review Board (IRB#: 2020002014). All respondents voluntarily and anonymously participated in the survey and were required to complete an online informed consent form prior to completing the survey in accordance with the relevant guidelines and regulations of the Rutgers Human Subjects Protection Program. The informed consent form included information, such as a summary of the research study, the estimated time to complete the survey, the benefits and risks of participating in the research study, data protection procedures, compensation details, and the contact information of the principal investigator (eg, the author) and the Rutgers University Human Subjects Protection Program. Respondents who completed the survey had the opportunity to enter a drawing to receive 1 of 75 available US $35 virtual gift cards. Additional information regarding ethical considerations and data protection is provided in Checklist 1.

### Measures

#### Overview

The cross-sectional online survey was administered using Qualtrics software (Qualtrics). The survey was developed in consultation with a tenured professor having expertise in conducting research on SRH among LBQ+ women of color and a professor having expertise in health informatics (see Acknowledgments). Before the launch of the survey, the initial survey instrument was tested with 4 individuals from the target population who completed the survey. This was done to culturally tailor the survey instrument [60]. The survey was revised following a focus group with the individuals who pilot tested the survey. The primary revisions to the survey following the pilot testing included providing a definition for chatbots, revising the wording of open-ended questions, and including discrimination measures reflective of the intersectional experiences of LBQ+ women of color. The individuals who pilot tested the survey did not participate in the study. The survey consisted of 88 questions. Survey domains included (1) sociodemographic characteristics, (2) mobile phone use, (3) chatbot use, (4) the acceptability of using digital health for sexual and reproductive health care, (5) SRH information-seeking behaviors, (6) awareness and attitudes about HIV prevention medications, (7) awareness and knowledge of HPV and cervical cancer prevention, (8) SRH access and use, and (9) discrimination in health care settings. The survey instrument is provided in Multimedia Appendix 1.

Most questions about sociodemographic characteristics, including gender identity, sexual orientation, race/ethnicity, and country and state of residence, were asked at the beginning of the survey to determine eligibility for participation in the research study. The race/ethnicity, sexual orientation, gender identity, age, and country of residence questions were used as screener questions to determine eligibility to continue the survey. Individuals who did not meet the eligibility criteria for the study were routed directly to the end of the survey. To prevent false responses from bots, indexing and multiple submissions were not allowed. One author (MT) reviewed the data weekly for suspicious responses. Individuals who passed the eligibility screening were informed that they could choose not to answer any questions on the survey and could discontinue their participation in the study at any time for any reason.

We aimed to assess the association between sociodemographic characteristics (ie, influencing factors) and the acceptability of using digital health modalities to support SRH information and service access and use among LBQ+ women of color. Considering that no previous literature exists regarding the potential relationship between these measures in the target population, a new a priori sample size analysis was conducted. Power calculations were performed for Fisher exact and multivariable logistic regression models using *α*=.05 and power=0.80 [75]. Assuming large effect sizes (p₀=0.30), the required sample size for our study was approximately 192. As the multivariable model included 5 predictors (ie, age, education, income, usual place of care, and health insurance), events per variable (EPV) adequacy was assessed [76]. Using a conservative threshold of 20 EPV, a minimum sample of 264 participants was required to ensure model stability. Therefore, the achieved sample size of 285 participants met both power and EPV requirements. Power estimates derived from the 2-group Fisher exact test also approximate the power of the logistic regression analyses, as both assess odds ratios (ORs) under similar assumptions; thus, the reported estimates apply to both unadjusted and adjusted models within this study design.

#### Mobile Phone and Chatbot Use

Mobile phone use was assessed with the following items adapted from the study by McCall et al [66]: (1) mobile phone ownership (yes or no), (2) the ability to send/receive text messages on a mobile phone (yes or no), (3) the frequency of sending SMS text messages (never, <1 time/week, 1‐6 times/week, 1‐3 times/day, and ≥4 times/day), (4) the use of mobile apps (yes or no), (5) the frequency of using a mobile phone to access mobile apps (never, <1 time/week, 1‐6 times/week, 1‐3 times/day, and ≥4 times/day), (6) the ability to complete video calls on a mobile phone (yes or no), and (7) the frequency of using a mobile phone to complete video calls (never, <1 time/week, 1‐6 times/week, 1‐3 times/day, and ≥4 times/day). Moreover, respondents were asked if they have used any of the following types of mobile apps to support their SRH: period tracking, patient portal, fertility/pregnancy planning, and birth control (yes or no).

Participants were provided with the following definition of a chatbot, adapted from the study by Palanica et al [77]: Chatbots, also known as conversational agents, are AI-enabled programs designed to imitate human conversations. Chatbots enable verbal and/or text communication with human users and can generate and retrieve information. Chatbots may be programmed to provide information relevant to specific user groups.

Chatbot use was assessed with the following questions: (1) “Have you ever used a chatbot or conversational agent for any purpose (even if unrelated to sexual and reproductive health)?” (2) “Have you ever sought sexual and reproductive health support from a chatbot or conversation agent?” (3) “In the past 12 months, have you sought sexual and reproductive health support from a chatbot or conversational agent?” and (4) “Would you be willing to use a chatbot to assist you with finding sexual and reproductive health information?”

#### Acceptability of Using Digital Health for SRH Information and Services

The acceptability of using digital health for sexual and reproductive health care access was measured by the comfortability of (1) communicating with a health care provider through video calls, mobile apps, or SMS text messaging to support access to SRH information and services and (2) using chatbots, SMS text messaging, or mobile apps to assess the risk for STIs and cervical cancer. Measures to assess the acceptability of using digital health, including chatbots for sexual and reproductive health care, were adapted from studies by McCall et al [66] and Nadarzynski et al [78]. Moreover, the following questions were asked to assess the acceptability of using digital health modalities: (1) “Would having the option to use a [chat bot/mobile app] to seek sexual and reproductive health information when you have an urgent health concern increase your comfort seeking sexual and reproductive services?” and (2) “Would having a [chatbot/mobile app] tailored to the information needs of LBQ+ women of color increase your comfort utilizing sexual and reproductive services?” Response options were yes or no.

At the end of the question block, respondents were asked the following questions: “Do you have any concerns about using a [mobile app/chatbot] to receive sexual and reproductive health information?” and “Do you have any concerns about using a [mobile app/video call/text message] to communicate with a health care provider about your sexual and reproductive health needs?” If they answered “yes” to these questions, they were presented with open-ended questions along with a text-entry box to make note of their concerns.

#### Awareness and Attitudes Toward HIV Prevention Medication

Participants were asked about their awareness and attitudes toward biomedical HIV prevention methods, including daily oral pre-exposure prophylaxis (PrEP) and the dapivirine (DAP) vaginal ring. Awareness of biomedical HIV prevention methods was assessed by asking the following two questions: (1) “Have you ever heard of PrEP (pre-exposure prophylaxis)? PrEP is when HIV-negative people take anti-HIV medications (antiretrovirals like Truvada) BEFORE HAVING SEX to prevent HIV” and (2) “Have you ever heard of dapivirine vaginal ring (also referred as the DAP ring)? The dapivirine vaginal ring is a form of pre-exposure prophylaxis. HIV-negative people with a vagina insert the ring which releases dapivirine (a topical antiretroviral) monthly to prevent HIV.” Response options to both questions were yes or no.

To assess attitudes toward the use of biomedical HIV prevention methods, participants were asked the following: “If a pill (drug/medication) that could be taken daily to prevent transmission of HIV from an HIV-positive sex partner to an HIV-negative partner were available I would take it” and “If a flexible, silicone ring that could be inserted into the vagina and provide sustained release of an anti-HIV drug monthly to prevent the transmission of HIV from an HIV-positive sex partner to an HIV-negative partner were available I would use it.” Response options were yes or no. Participants who selected no were asked the following question: “Why would you not take the pill/use the ring?” Response options included “I’m not at risk of HIV infection,” “I would not want to pay for it,” “I would be afraid that someone would find out that I was taking it,” “I’m afraid of potential side effects,” “I don’t like taking pills daily,” “I don’t like having objects inserted in my vagina,” and “I don’t believe it would work.”

#### HPV and Cervical Cancer Awareness and Knowledge

Respondents’ knowledge and awareness of HPV and cervical cancer were measured using questions adapted from a survey instrument developed by Lyson et al [79]. HPV and HPV prevention awareness were assessed by asking four questions: (1) “Have you ever heard of HPV? HPV stands for Human Papillomavirus?” (2) “Have you ever heard of the HPV vaccine or shots to prevent cervical cancer?” (3) “Have you ever heard of an HPV test?” and (4) “Have you ever heard of a Pap test?”

Moreover, eight questions assessed knowledge of HPV and cervical cancer prevention: (1) “Do you think HPV can cause cervical cancer?” (2) “Do you think you can get HPV through sexual contact?” (3) “Do you think HPV causes AIDS?” (4) “Do you think HPV can go away on its own without treatment?” (5) “Do you think an HPV test can detect cervical cancer?” (6) “Do you think you think you need a Pap test in order to receive a cervical cancer diagnosis?” (7) “Do you think you only need one dose of the HPV vaccine?” and (8) “Do you think you can get the HPV vaccine if you’re over the age of 45?” Response options were yes or no.

### Data Analysis

#### Quantitative Data Analysis

Data were analyzed using descriptive statistics (ie, means, percentages, frequencies, and SDs) to describe sociodemographic characteristics, characterize attitudes toward STIs and cervical cancer prevention methods, and assess the acceptability and concerns of using digital health modalities to promote access to SRH information and services among LBQ+ women of color in the United States. Sociodemographic characteristics included race/ethnicity (American Indian, Asian, Black/African American, Hispanic/Latina, Middle Eastern/North African, Native Hawaiian, or other Pacific Islander), age, education (high school diploma or General Educational Development, some college or associate’s degree, bachelor’s degree, or master’s degree or higher), sexual orientation (lesbian, bisexual, pansexual, queer, or another sexual identity), income (≤US $10,000, US $10,000-$24,999, US $25,000-$49,999, US $50,000-$100,000, or ≥US $100,000), region of residence in the United States (Midwest, Northeast, South, or West), health insurance (uninsured or insured), and usual place of care (yes or no). Additionally, respondents had the option to select more than one race/ethnic category. Individuals who selected more than one option were categorized as multiracial. The age variable was grouped (18‐20, 21‐29, 30‐45, 46‐65, or >65 years) according to guidelines by the US Preventive Services Task Force that recommend cervical cancer screening every 3 years for individuals with vaginas who are aged 21‐29 years and every 3‐5 years for individuals with vaginas who are aged 30‐65 years based on the test. This age grouping is also based on the results from a previous cross-sectional survey study of health care discrimination and Pap test use among Black LBQ+ individuals assigned female at birth [15]. Age was dichotomized into 2 categories (<45 years and ≥45 years), education was dichotomized into 2 categories (less than a bachelor’s degree and a bachelor’s degree or higher), and income was dichotomized into 2 categories (<US $49,000 and ≥US $50,000).

The Fisher exact test was used to assess whether a statistically significant association exists between comfortability with the use of each digital health modality (eg, video calls, SMS text messaging, and mobile apps) to communicate with a health care provider to receive support in using SRH services (indicating agreement vs not indicating agreement) and education level, age, income, usual place of care, or health insurance. Multivariable logistic regression modeling was performed to estimate ORs and their corresponding CIs to assess the strength of the association between comfortability with the use of each digital health modality to communicate with a health care provider to receive support in using SRH services (indicating agreement vs not indicating agreement) and age, education level, income, health insurance, usual place of care, and region of residence as explanatory variables. Response options were dichotomized into those indicating agreement (agree or somewhat agree) and those not indicating agreement (undecided, somewhat disagree, or disagree). Statistical significance was determined at a *P* value of .05 for all tests. No adjustments were made for multiple comparisons.

Multivariable logistic regression modeling estimated adjusted odds ratios (aORs) and 95% CIs to assess the association between comfortability with the use of each digital health modality to communicate with a health care provider to receive support in using SRH services (indicating agreement vs not indicating agreement) and each of the 4 HPV awareness outcomes (eg, heard of HPV, HPV vaccine, HPV test, and Pap test). To assess the association between comfortability with the use of each digital health modality to communicate with a health care provider to receive support in using SRH services (indicating agreement vs not indicating agreement) and HPV knowledge, we used 2 complementary regression approaches consistent with the best practices for analyzing ordinal and continuous knowledge outcomes [80-82]. HPV knowledge was dichotomized into 2 categories: high HPV knowledge (≥4 correct responses) and low HPV knowledge (<4 correct responses). A multivariable logistic regression model was fit to estimate aORs and 95% CIs for the association between comfortability with the use of each digital health modality and the likelihood of respondents having high HPV knowledge. To complement the binary model and capture variability in knowledge scores, the continuous HPV knowledge score (range 0‐8) was analyzed using multivariable linear regression. This model estimated adjusted β coefficients and corresponding 95% CIs to assess the relationship between digital health modality comfortability and incremental changes in HPV knowledge. Both models were adjusted for sociodemographic characteristics selected a priori based on their relevance to HPV awareness and knowledge (ie, age, education, income, health insurance status, and usual place of care). Statistical significance was determined at a *P* value of <.05 for both tests. Statistical analyses were performed using SPSS version 28 (IBM Corp).

#### Qualitative Data Analysis

Survey respondents who indicated that they had concerns using digital health modalities to receive SRH information or to communicate with a health care provider about their SRH were asked to share their concerns through open-ended questions. There was no character limit on the open-ended response field. Responses to the questions, “What are your concerns about using a [mobile app/chatbot/text message] to receive sexual and reproductive health information?” and “What are your concerns about using a [mobile app/video call/text message] to communicate with a health care provider about your sexual and reproductive health needs?” were imported into NVivo 13 software (QSR International) for thematic analysis [83]. Using thematic analysis methods [83,84], the first author (MT; an experienced qualitative and mixed-methods researcher with a PhD in Information Science) familiarized herself with the data by reading through each response twice and taking notes while developing initial open codes [85]. After rereading the responses, MT developed in vivo codes to capture the respondents’ perspectives and to ensure the confirmability of the analysis [86]. During second-cycle coding, the initial and in vivo codes were merged into high-level themes. Saturation was reached once no new themes emerged from the data analysis [87]. To enhance the reliability of the analysis, the coding process and results of the thematic analysis were reviewed and discussed during peer debriefing with a senior faculty colleague with expertise in health informatics. To further bolster the credibility of the thematic analysis, the results were member checked by survey respondents.

## Results

### Participants

From November 2020 to March 2021, a total of 350 respondents were deemed eligible to participate in the study. These respondents completed the informed consent process and the first page of the survey. Of the 350 respondents, 285 (81.4% completion rate) completed the survey. On average, it took respondents 22.7 minutes to complete the survey. Table 1 summarizes the sociodemographic characteristics of the LBQ+ women of color who completed the survey.

**Table 1.** Sociodemographic characteristics of the survey respondents.

Characteristic		Value (N=285), n (%)	
Age range (years)			
18‐20		72 (25.3)	
21‐29		129 (45.3)	
30‐45		67 (23.5)	
46‐65		14 (4.9)	
>65		3 (1.1)	
Race/ethnicity			
American Indian		9 (3.2)	
Asian		43 (15.1)	
Black or African American		145 (50.1)	
Hispanic or Latina		63 (22.1)	
Multiracial		25 (8.8)	
Sexual orientation identity			
Lesbian		76 (26.6)	
Bisexual		174 (61.1)	
Pansexual		23 (8.1)	
Queer		9 (3.2)	
Another sexual identity		3 (1.1)	
Educational attainment			
High school diploma or GED^a^		48 (16.8)	
Some college or associate’s degree		137 (48.1)	
Bachelor’s degree		63 (22.1)	
Master’s degree or above		37 (13.0)	
Income (US$)^b^			
<10,000		2 (0.7)	
10,000-24,999		15 (5.3)	
25,000-49,999		106 (37.2)	
50,000-100,000		134 (47.0)	
>100,000		28 (9.8)	
Geographic region			
Midwest		43 (15.1)	
Northeast		119 (41.8)	
South		86 (30.2)	
West		37 (13.0)	
Health insurance status			
Uninsured		72 (25.3)	
Insured		213 (74.7)	
Usual place of care			
Yes		178 (62.4)	
No		107 (37.6)	

a GED: General Educational Development tests.

b The total sample size is 285, and percentages may not sum up to 100% because of item missingness (n=2) and rounding.

Respondents were primarily Black or African American (145/285, 50.1%), mainly identified as bisexual (145/285, 50.1%), and ranged in age from 18 to 72 years (mean age 28.29, SD 10.38 years). Most respondents lived in the Northeastern United States (119/285, 41.8%) and had some college or associate’s degree (137/285, 48.1%). The annual household income was reported to be US $25,000-$49,999 for 37.2% (106/285) of respondents and US $50,000-$100,000 for 47.0% (134/285) of respondents. Most respondents reported having health insurance (213/285, 74.7%) and a usual place of care (178/285, 62.4%).

### Mobile Phone and Chatbot Use

Table 2 presents the mobile phone use of survey respondents. All participants (285/285, 100%) reported owning a mobile phone and being able to send SMS text messages. Most respondents indicated texting ≥1 time per day (231/285, 93.4%) and mentioned that their phones had video call capability (207/285, 72.6%). Most respondents who indicated that their phone had video call capability reported making video calls at least 1‐6 times per week (83/207, 40.1%). Most respondents (172/285, 60.4%) reported ever using a menstrual cycle or fertility tracking app during their lifetime, and more than half (182/285, 63.9%) indicated using a patient portal app during their lifetime. The majority of respondents (203/285, 71.2%) accessed mobile apps on their phone 1‐3 times per day. Most respondents (208/285, 73.0%) had not used a pregnancy tracking app, and more than half (159/285, 55.8%) had not used a birth control app. Nearly half of the respondents (133/285, 46.7%) reported using a chatbot for any purpose, and a few respondents (51/285, 17.9%) had used a chatbot to obtain SRH information. Among respondents who had ever used a chatbot to obtain SRH information, most (37/51, 72.5%) had not used one in the past 12 months for that purpose.

**Table 2.** Mobile phone use characteristics.

Characteristic		Value (N=285), n (%)	
Mobile phone ownership			
Yes		285 (100)	
No		0 (0)	
Mobile phone text message capability			
Yes		285 (100)	
No		0 (0)	
Frequency of sending SMS text messages^a^			
Never		5 (1.8)	
<1 time/week		12 (4.2)	
1‐6 times/week		35 (12.3)	
1‐3 times/day		63 (22.1)	
≥4 times/day		168 (59.0)	
Mobile phone video capability			
Yes		207 (72.6)	
No		78 (27.4)	
Frequency of video call use^b^			
Never		15 (7.2)	
<1 time/week		39 (18.8)	
1‐6 times/week		83 (40.1)	
1‐3 times/day		23 (11.1)	
≥4 times/day		47 (22.7)	
Frequency of accessing mobile apps			
Never		8 (2.8)	
<1 time/week		15 (5.3)	
1‐6 times/week		40 (14.0)	
1‐3 times/day		203 (71.2)	
≥4 times/day		19 (6.7)	
Use of a period, fertility, or menstrual cycle tracking mobile app (lifetime)			
Yes		172 (60.4)	
No		83 (29.1)	
Prefer not to say		30 (10.5)	
Use of a patient portal, MyChart mobile app (lifetime)			
Yes		182 (63.9)	
No		103 (36.1)	
Use of a pregnancy tracking app^c^ (lifetime)			
Yes		45 (15.8)	
No		208 (73.0)	
Prefer not to say		22 (7.7)	
Use of a birth control app (lifetime)			
Yes		114 (40.0)	
No		159 (55.8)	
Prefer not to say		12 (4.2)	

a The total sample size is 285, and percentages may not sum up to 100% because of item missingness (n=2) and rounding.

b Respondents who indicated that they did not have video call capability (n=78) were not presented with the frequency of use question.

c The total sample size is 285, and percentages may not sum up to 100% because of item missingness (n=10) and rounding.

### Awareness and Attitudes Toward HIV Prevention Medication

While 61.8% (176/285) of respondents were aware of PrEP (when taken as a daily pill to prevent HIV transmission from an HIV-positive partner to an HIV-negative partner), most respondents (262/285, 91.9%) had not heard of the DAP ring, a vaginal insert, to prevent HIV during vaginal sex. Comfortability (agree or somewhat agree) with the use of video calls, SMS text messaging, and mobile apps to communicate with a health care provider to receive support in using SRH services was not significantly associated with awareness of PrEP or the DAP ring (all *P*≥.05). Sociodemographic covariates were also not significantly associated with awareness of HIV prevention methods (ie, DAP ring and PrEP).

About half (154/285, 54.0%) of respondents mentioned that they would be willing to take PrEP as a daily pill to prevent acquiring HIV. There were far less favorable attitudes toward the DAP ring, with 93.7% (267/285) reporting that they would not use it as a form of HIV prevention. The top three reasons respondents indicated that they would not take PrEP as a daily pill were: (1) “I’m not at risk of HIV infection” (123/154, 79.9%), (2) “I’m afraid of potential side effects” (132/154, 85.7%), and (3) “I would not want to pay for it” (50/154, 32.5%). Similarly, the top three reasons respondents indicated that they would not use a DAP ring for HIV prevention were: (1) “I’m not at risk for HIV infection” (128/154, 83.1%), (2) “I’m afraid of potential side effects” (119/154, 77.3%), and (3) “I don’t like having objects inserted in my vagina” (91/154, 59.1%). Comfortability with the use of video calls, SMS text messaging, and mobile apps was not significantly associated with willingness to take PrEP (all *P*≥.05). No sociodemographic covariates demonstrated significant associations with this outcome. Higher income was significantly associated with lower willingness to use the DAP ring (aOR 0.19, 95% CI 0.05‐0.78; *P*=.02). No other demographic covariates or digital health modality comfortability variables were significantly associated with willingness to use the DAP ring (all *P*≥.05).

### HPV and Cervical Cancer Awareness and Knowledge

Overall, awareness about HPV was high in the sample (213/285, 74.7%) (Table 3). Most respondents were aware of HPV screening measures, including an HPV test (193/285, 67.7%) and a Pap test (247/285, 86.7%). However, only 37.9% (108/285) of respondents had awareness of the HPV vaccine. Comfortability (agree or somewhat agree) with the use of mobile apps to communicate with a health care provider to receive support in using SRH services was significantly associated with greater odds of HPV vaccine awareness (aOR 1.91, 95% CI 1.12‐3.26; *P*=.02). There were no statistically significant associations between the use of SMS text messaging, video calls, or mobile apps and awareness of HPV, HPV testing, or Pap testing. Comfort with using video calls and SMS text messaging was not significantly associated with any HPV awareness outcome. A knowledge score was calculated for each respondent. The knowledge score was the total number of correct responses to the 8 knowledge questions. Respondents who answered a question incorrectly or selected “don’t know” were given a score of zero. Knowledge of HPV and cervical cancer prevention was low in the sample. The mean knowledge score was 3.78 (SD 1.22). Approximately 52.7% (1202/2280) of all knowledge item responses were incorrect. In the multivariable logistic regression model using the dichotomous HPV knowledge variable (high HPV knowledge, ≥4 correct responses; low HPV knowledge, <4 correct responses), comfortability with the use of video calls, SMS text messaging, or mobile apps was not significantly associated with high HPV knowledge (all *P*>.10). aORs for all 3 digital health modalities were nonsignificant (all *P*>.10). None of the sociodemographic covariates were significantly associated with high HPV knowledge (all *P*>.10). In the multivariable linear regression model using the continuous HPV knowledge score as the dependent variable, there was no statistically significant association between comfortability with the use of video calls, SMS text messaging, or mobile apps and the HPV knowledge score (all *P*>.10). None of the sociodemographic covariates were significantly associated with the continuous HPV knowledge score (all *P*>.10).

**Table 3.** Human papillomavirus and cervical cancer awareness and knowledge (N=285).

Characteristic	Yes, n (%)	No, n (%)
Awareness of HPV^a^ (prevention)		
Ever heard of HPV	213 (74.7)	72 (25.3)
Ever heard of the HPV vaccine	108 (37.9)	177 (62.1)
Ever heard of an HPV test	193 (67.7)	92 (32.3)
Ever heard of a Pap test	247 (86.7)	38 (13.3)
HPV knowledge		
HPV causes cervical cancer^b^	233 (81.8)	42 (14.7)
Get HPV from sexual contact^b^	253 (88.8)	26 (9.1)
HPV causes AIDS^b^	25 (8.8)	256 (89.8)
HPV can go away without treatment	12 (4.2)	273 (95.8)
HPV test can detect cervical cancer	263 (92.3)	22 (7.7)
Pap test needed for cervical cancer diagnosis^b^	165 (57.9)	108 (37.9)
Only need 1 dose of the HPV vaccine^b^	186 (65.3)	75 (26.3)
Can get the HPV vaccine over the age of 45^b^	216 (75.8)	62 (21.8)

a HPV: human papillomavirus.

b The total sample size is 285, and percentages may not sum up to 100% because of respondents answering “don’t know” (n=1‐24).

### The Acceptability of Digital Health to Support SRH Information and Service Access

Most LBQ+ women of color in the sample agreed with the use of video calls to communicate with a health care provider (233/285, 81.8%) to receive support in using SRH services, followed by mobile apps (175/285, 61.4%) and SMS text messaging (123/285, 43.2%). Statistically significant associations were found between education level (less than a bachelor’s degree versus a bachelor’s degree or higher) and comfortability (agree or somewhat agree) with the use of video calls and mobile apps to communicate with a health care provider to receive support in using SRH services (all *P*<.001). Respondents with a bachelor’s degree or higher were significantly more likely than respondents with less than a bachelor’s degree to agree with the use of video calls or mobile apps to communicate with a health care provider to receive support in using SRH services (Table 4).

**Table 4.** Agreement with the use of each digital health modality to communicate with a health care provider to receive support in using sexual and reproductive health information and services by participant characteristics (N=285).

Characteristic, modality, and category	Agreed, n	Did not agree^a^, n	Fisher exact *P* value^b^	Odds ratio (95% CI)^b^	
Education					
Video call			<.001	0.00 (0.00‐0.24)	
<Bachelor’s degree (n=185)	133	52			
≥Bachelor’s degree (n=100)	100	0			
SMS text messaging			>.99	1.02 (0.61‐1.71)	
<Bachelor’s degree (n=185)	80	105			
≥Bachelor’s degree (n=100)	43	57			
Mobile app			<.001	0.41 (0.23‐0.74)	
<Bachelor’s degree (n=185)	101	84			
≥Bachelor’s degree (n=100)	74	26			
Age					
Video call			.59	0.79 (0.35‐1.79)	
<45 years (n=221)	179	42			
≥45 years (n=64)	54	10			
SMS text messaging			<.001	0.11 (0.14‐0.53)	
<45 years (n=221)	71	150			
≥45 years (n=64)	52	12			
Mobile app			<.001	6.27 (0.07‐0.25)	
<45 years (n=221)	157	64			
≥45 years (n=64)	18	46			
Income					
Video call			.88	0.95 (0.50‐1.83)	
<US $49,000 (n=123)	100	23			
≥US $50,000 (n=162)	133	29			
SMS text messaging			<.001	2.26 (1.32‐3.93)	
<US $49,000 (n=123)	67	56			
≥US $50,000 (n=162)	56	106			
Mobile app			.87	0.96 (0.58‐1.58)	
<US $49,000 (n=123)	77	46			
≥US $50,000 (n=162)	98	64			
Usual place of care					
Video call			<.001	0.17 (0.07‐0.43)	
Usual place (n=178)	132	46			
No usual place (n=107)	101	6			
SMS text messaging			.71	0.90 (0.53‐1.50)	
Usual place (n=178)	75	103			
No usual place (n=107)	48	59			
Mobile app			.71	1.11 (0.67‐1.94)	
Usual place (n=178)	111	67			
No usual place (n=107)	64	43			
Health insurance status					
Video call			<.001	239.8 (56.1‐1025.7)	
Insured (n=213)	211	2			
Uninsured (n=72)	22	50			
SMS text messaging			<.001	0.02 (0.01‐0.06)	
Insured (n=213)	55	158			
Uninsured (n=72)	68	4			
Mobile app			<.001	6.34 (3.23‐12.45)	
Insured (n=213)	154	59			
Uninsured (n=72)	21	51			

a The “did not agree” group was created by combining “undecided” responses with “somewhat disagree” and “disagree” responses.

b Comparison between characteristic categories.

Statistically significant associations were also found between age (<45 years vs ≥45 years) and comfortability (agree or somewhat agree) with the use of mobile apps and SMS text messaging to communicate with a health care provider to receive support in using SRH services (all *P*<.001). Respondents aged <45 years were more than 6 times more likely to agree with the use of mobile apps than those aged ≥45 years. Moreover, respondents aged ≥45 years were significantly more likely to agree with the use of SMS text messaging than those aged <45 years (Table 4).

Statistically significant associations were found between income (<US $49,000 vs ≥US $50,000) and comfortability (agree or somewhat agree) with the use of SMS text messaging (67/125 agreed vs 133/162 agreed, respectively; *P*<.001). Women with an income of <US $49,000 were more than 2 times more likely than those with an income of ≥US $50,000 to agree with the use of SMS text messaging (Table 4).

Statistically significant associations were found between usual place of care (usual place of care vs no usual place of care) and comfortability (agree or somewhat agree) with the use of video calls (132/178 agreed vs 101/107 agreed, respectively; *P*<.001). Women without a usual place of care were significantly more likely than those with a usual place of care to agree with the use of video calls (Table 4).

Statistically significant associations were found between health insurance status (insured vs uninsured) and comfortability (agree or somewhat agree) with the use of video calls (211/213 agreed vs 22/72 agreed, respectively; *P*<.001), SMS text messaging (55/213 agreed vs 68/72 agreed, respectively; *P*<.001), and mobile apps (154/213 agreed vs 21/72 agreed, respectively; *P*<.001). Women with health insurance were significantly more likely than those without health insurance to agree with the use of video calls and mobile apps. Women without health insurance were far more likely than those with insurance to agree with the use of SMS text messaging (Table 4).

There were no statistically significant associations between education level (less than a bachelor’s degree vs a bachelor’s degree or higher) and comfortability (agree or somewhat agree) with the use of SMS text messaging, between age (<45 years vs ≥45 years) and comfortability with the use of video calls, between income (<US $49,000 vs ≥US $50,000) and comfortability with the use of video calls and mobile apps, and between usual place of care (usual place of care vs no usual place of care) and comfortability with the use of SMS text messaging and mobile apps (all *P*<.05) (Table 4).

The results of the multivariable logistic regression modeling showed no statistically significant ORs (all *P*>.05) between respondents who agreed to use mobile apps (Table 5) or SMS text messaging (Multimedia Appendix 2) to communicate with a health care provider to receive support in using SRH services and age, education level, income, health insurance, and usual place of care. Women in the Midwest had significantly lower odds of agreeing with the use of video calls compared with those in the Northeast (aOR 0.37, 95% CI 0.14-0.98; *P*=.045) (Multimedia Appendix 3).

**Table 5.** Multivariable logistic regression models for comfortability with the use of mobile apps to communicate with a health care provider to receive support in using sexual and reproductive health information and services (N=285).

Category		Agreed, n	Did not agree, n	Adjusted OR^a^ (95% CI)	*P* value
Age (years)	
<45 (reference)		157	64	1.00	—^b^
≥45		18	46	4.36 (0.74‐25.7)	.10
Income (US$)	
≥50,000 (reference)		98	64	1.00	—
<49,000		77	46	0.69 (0.30‐1.59)	.38
Education	
<Bachelor’s degree (reference)		101	84	1.00	—
≥Bachelor’s degree		74	26	0.26 (0.04‐1.58)	.14
Usual place of care	
Yes (reference)		111	67	1.00	—
No		64	43	1.50 (0.22‐10.3)	.68
Insurance	
Insured (reference)		154	59	1.00	—
Uninsured		21	51	0.52 (0.09‐3.03)	.46
Region	
Northeast (reference)		79	40	1.00	—
Midwest		22	21	0.60 (0.28‐1.26)	.18
South		44	42	0.65 (0.19‐2.24)	.50
West		30	7	1.93 (0.36‐10.2)	.44

a OR: odds ratio.

b Not applicable.

**Figure 1.**
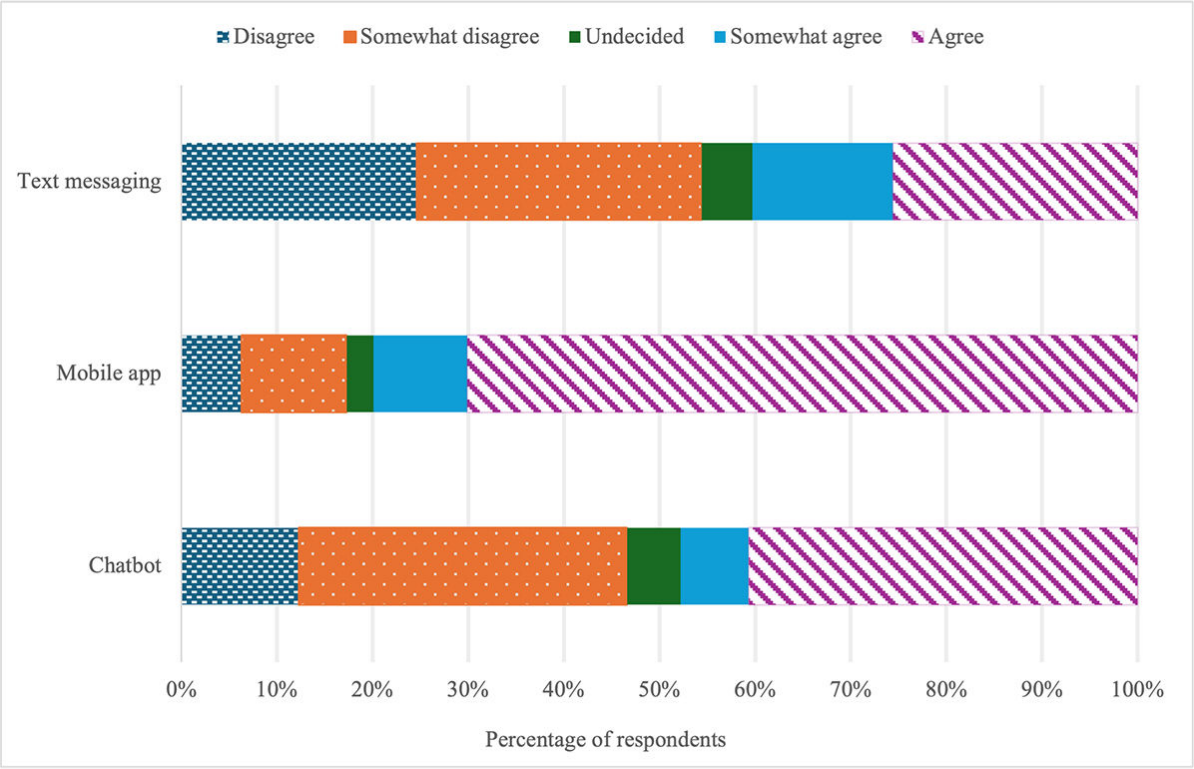
Sample percentages by modality for responses to the statement, “I would feel comfortable using a [modality] to assess my risk for cervical cancer.”

Survey results revealed that nearly half of the respondents (136/285, 47.7%) agreed that they would be comfortable using a chatbot to self-assess the risk for cervical cancer. In comparison, almost half of all respondents (133/285, 46.7%) indicated disagreement that they would be comfortable using a chatbot to self-assess the risk for cervical cancer. There were more favorable views toward using mobile apps (228/285, 80.0%) for self-assessing the risk for cervical cancer. Most respondents (155/285, 54.4%) disagreed that they would be comfortable with using text messaging to self-assess the risk for cervical cancer (Figure 1).

**Figure 2.**
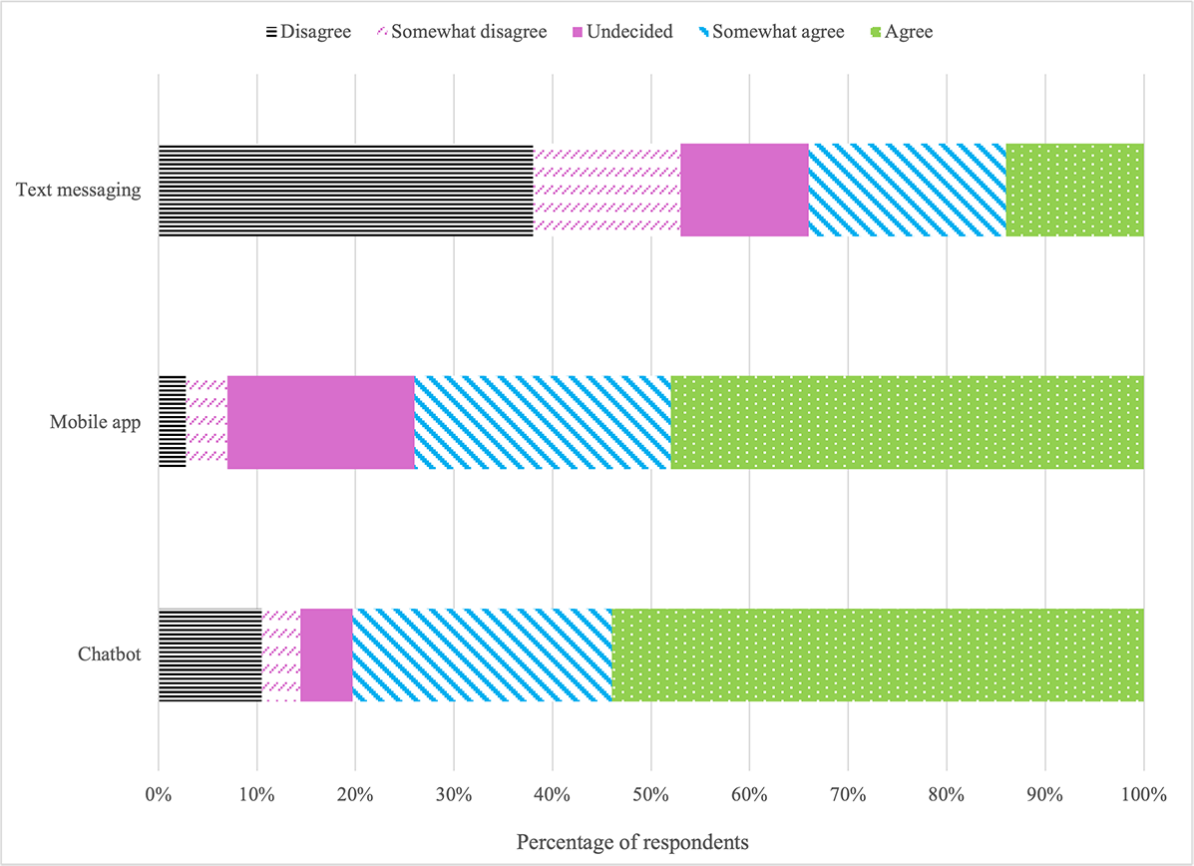
Sample percentages by modality for responses to the statement, “I would feel comfortable using a [modality] to assess the risk for acquiring sexually transmitted infections (eg, HPV, gonorrhea, and chlamydia).”

The majority of respondents (229/285, 80.3%) were comfortable with using a chatbot to self-assess the risk for STIs (eg, HIV, HPV, and gonorrhea). On the other hand, 14.4% (41/285) of respondents were uncomfortable using a chatbot to self-assess the risk for STIs, while 5.3% (15/285) were undecided. Moreover, 74.0% (211/285) of respondents agreed that they would be comfortable using mobile apps, and 34.0% (97/285) agreed that they would be comfortable using text messaging to self-assess the risk for STIs (Figure 2).

Most respondents (243/285, 85.3%) indicated that having mobile apps tailored to the information needs of LBQ+ women of color would increase their comfort in using SRH services, and fewer respondents (180/285, 63.2%) reported that a chatbot tailored to the information needs of LBQ+ women of color would increase their comfort in using SRH; however, there was still high support for this modality.

### Qualitative Results

#### Overview

Respondents were asked if they had concerns about using chatbots or mobile apps to receive SRH information. Among the respondents, 54.0% (154/285) indicated concerns about using a chatbot, and 48.1% (137/285) indicated concerns about using mobile apps to receive SRH information. Fewer participants indicated concerns about using digital health modalities to communicate with a health care provider. Among the respondents, 12.3% (35/285) reported concerns with using SMS text messaging, and approximately 5% reported concerns with using mobile apps (14/285, 4.9%) or video calls (14/285, 4.9%) to communicate with a health care provider. Thematic analysis revealed the following key thematic concerns: data privacy and confidentiality, lack of affective communication (eg, emotions and feelings), and technology connectivity and adoption issues. Each theme has been discussed below.

#### Data Privacy and Confidentiality

The first theme, *data privacy and confidentiality*, describes respondents’ concerns about who would have access to the data and information they share through digital health modalities, as well as the potential harms resulting from unauthorized parties accessing their sensitive information. Respondents acknowledged the sensitive nature of SRH information and emphasized the need for transparency in data storage, use, and access. The potential risk of data misuse and the fear of having their SRH history and sexuality made public were also concerns among the respondents. These concerns were related to using chatbots and mobile apps to share SRH information and communicate with a health care provider. Several respondents noted that they did not have a private place to speak with a health care provider via a video call about SRH services. Illustrative quotes from the thematic analysis are as follows:

Who will have access to my stuff? What will happen to it once I close the window? I don’t want this kind of stuff being hacked. I need it to be safe.

I like the option to chat on video, but I wouldn’t want the wrong person hear me talk about this stuff.

Don’t want my STD treatment showing up in ads on my phone now. Or it being linked to an account I didn’t give it access to.

#### Lack of Affective Communication

The second theme, *lack of affective communication*, entails respondents’ concerns that obtaining SRH information from a chatbot or mobile app would be impersonal. They were skeptical of the chatbot’s ability to imitate a human’s affective (eg, emotions and feelings) traits. They also expressed concerns about the generic communication style of chatbots. The lack of empathy conveyed when communicating SRH information through a chatbot or mobile app was a concern among respondents. Similarly, communicating with a health care provider via text messaging was described as “impersonal” among respondents. Respondents were concerned about losing the nuances of face-to-face verbal communication. Illustrative quotes from the thematic analysis are as follows:

Chatbot would be dry. It won’t know how I’m feeling in the moment.

With text, you can’t always read emotion. I need to know that they care.

Just an impersonal feeling. It can give me the information, but can it communicate?

No empathy or understanding of what I’m going through.

#### Technology Connectivity and Adoption

The final theme, *technology connectivity and adoption*, describes technology connectivity issues and a lack of experience using digital health modalities as barriers to their use to obtain SRH information or communicate with a health care provider. Respondents anticipated problems with internet access, device malfunctions, or issues with the digital health modality (eg, mobile app and chatbot) used to obtain SRH information or services. Some of the concerns included an application crash, a chatbot not working, a video call dropping, or a text message not being delivered. There were concerns that they did not have the digital literacy skills necessary to use specific digital health modalities (eg, What kinds of questions to ask a chatbot? How to use a particular mobile app?). Illustrative quotes from the thematic analysis are as follows:

I might lose wi-fi during a call.

The text might not go through, or it might go to the wrong person.

I’m not sure about how to use it. Technology can be great, but you can’t assume people will know what to do.

## Discussion

### Principal Findings

This study investigated the awareness, knowledge, and attitudes of LBQ+ women of color in the United States toward STIs and cervical cancer prevention methods and the acceptability and concerns of using digital health, including telemedicine, mobile health (mHealth), and AI-enabled chatbots, to promote sexual and reproductive health care access. The results of this study provide new empirical contributions regarding attitudes toward STIs (eg, HPV and HIV) and cervical cancer prevention methods among LBQ+ women of color. While there has been extensive research regarding PrEP awareness among sexual minority men in the United States, there is no such study exploring its awareness among LBQ+ women of color [88]. More than half of the respondents (176/285, 61.8%) were aware of PrEP when taken as a daily pill to prevent HIV transmission from an HIV-positive partner to an HIV-negative partner. Extant literature reports low PrEP awareness among cisgender Black heterosexual women in the United States [89-91]. These results may indicate that LBQ+ women of color have greater awareness of PrEP than their heterosexual counterparts. About half of the respondents (154/285, 54.0%) indicated they would be willing to take PrEP. Respondents who were not willing to take PrEP reported a belief that they were not at risk for acquiring HIV and a fear of the potential side effects of the medication as the primary reasons. These findings are consistent with research literature among cisgender heterosexual women of color in the United States who report being less willing to take PrEP due to their low perception of HIV risk [91,92], with 50.1% (145/285) of the sample being comprised of cisgender Black women. Given that cisgender Black women accounted for nearly 50% of new HIV diagnoses among US women in 2022 while comprising less than 15% of the population of women, there is a need to increase the accuracy of HIV risk awareness and awareness of PrEP as an HIV prevention method among LBQ+ women of color [93].

There was high awareness about HPV (213/285, 74.7%) and screening methods for HPV and cervical cancer, including the HPV test (193/285, 67.7%) and the Pap test (247/285, 86.7%) among respondents. However, there was low awareness of the HPV vaccine (108/285, 37.9%) and low knowledge about cervical cancer prevention and HPV. For example, more than half of the respondents (186/285, 65.3%) answered that only 1 dose of the HPV vaccine is needed. Additionally, only 4.2% (12/285) of participants answered that HPV can go away without treatment. Extant literature reports high awareness of the HPV vaccine among LBQ+ women and high awareness of HPV among Black and Latina women in the United States [35,94,95]. The results indicate that there is a need to improve the knowledge and awareness of cervical cancer prevention and HPV prevention among LBQ+ women of color. The findings from this study suggest that LBQ+ women of color may not perceive themselves to be at risk for STIs such as HPV and HIV. Future work should focus on the development of resources, tools, and interventions aimed at improving HPV and cervical cancer awareness and knowledge among LBQ+ women of color.

All respondents owned a mobile phone that could send SMS text messages, and most of their phones had video call capability (207/285, 72.6%). While there is scarce literature regarding mobile technology ownership among LBQ+ women of color, this finding is consistent with explorations among similar populations (eg, heterosexual women of color and LBQ+ women) [65,66]. Most studies exploring mobile app usage among LBQ+ women have focused on their use of dating apps for partner-seeking [96,97]. Findings from this study suggest that the use of mobile phones to deliver health interventions is acceptable among LBQ+ women of color. Our study found that most respondents (172/285, 60.4%) had experience using a menstrual or fertility tracking app on their mobile phone at some point in their lifetime, and more than half (182/285, 63.9%) had used a patient portal app on their mobile phone at some point in their lifetime. This finding provides new empirical insights about the use of mHealth among LBQ+ women of color for their sexual and reproductive health care. It also contributes to the limited but growing body of research literature that explores the sexual and reproductive health care experiences of LBQ+ women of color [28,98-100].

There was high comfortability (agreement) with the use of video calls (233/285, 81.8%) and mobile apps (175/285, 61.4%) to communicate with a health care provider to receive support in using SRH services among respondents. Respondents with a bachelor’s degree or higher were significantly more likely to agree with the use of video calls or mobile apps to communicate with a health care provider to receive support in using SRH services than respondents with less than a bachelor’s degree. The results are consistent with previous research, which reports that individuals with higher educational levels are significantly more likely to use video calls for health care visits compared to those with lower educational attainment [101,102]. Significant findings emerged regarding age. Respondents aged <45 years were more likely to agree with the use of mobile apps to communicate with a health care provider to receive support in using SRH services than those aged ≥45 years, while respondents aged ≥45 years were significantly more likely to agree with the use of SMS text messaging than those aged <45 years. This finding may be attributed to research indicating that mHealth adoption, particularly the use of mobile apps, is slower among older populations. The National Poll on Healthy Aging found that among 2110 US adults aged 50‐80 years, 56% reported never using mobile apps for health-related purposes [103]. Aging populations may be more adept at SMS text messaging as a digital literacy skill as opposed to using mobile apps [104].

The results revealed that respondents with health insurance were significantly more likely than those without health insurance to agree with the use of video calls and mobile apps to communicate with a health care provider and receive support in using SRH services. Additionally, LBQ+ women of color in the sample with a usual place of care were significantly more likely to agree with the use of video calls than those without a usual place of care. These results indicate that social determinants of health (ie, health insurance and usual place of care) may influence comfortability with the use of digital health modalities for accessing SRH services [105]. Addressing social determinants that may limit access to SRH services and comfortability with the uptake of digital health modalities should be a key goal of future informatics interventions designed to advance sexual and reproductive health care equity among LBQ+ women of color.

While only half of the respondents (133/285, 46.7%) reported past use of a chatbot for any purpose, most respondents (229/285, 80.4%) indicated that they would be comfortable using a chatbot to self-assess the risk for STIs. However, there was less favorable comfortability with the use of a chatbot to self-assess the risk for cervical cancer (136/285, 47.7% indicated agreement). The results demonstrated that most respondents agreed that a chatbot (180/285, 63.2%) or mobile app (243/285, 85.3%) tailored to the SRH information needs of LBQ+ women of color would increase their comfort in accessing SRH information and services. These findings are consistent with results from a study assessing attitudes toward AI among marginalized communities, which found that racial minorities had more positive attitudes toward AI than other groups [106]. These findings indicate that leveraging chatbots may help address the barriers to accessing sexual and reproductive health care that LBQ+ women of color face, such as discrimination in health care settings and patient-provider communication, by providing nonjudgmental, nonstigmatizing information that centers their information needs and cultural norms in a safe environment [39].

Some respondents reported concerns about data privacy and confidentiality, lack of affective communication (when using a chatbot or communicating with a health care provider via SMS text messaging), and technology connectivity and adoption issues. All these concerns may pose challenges to the adoption and sustained use of digital health modalities to support sexual and reproductive health care access among LBQ+ women of color, especially impersonal or detached communication when using a chatbot to obtain SRH information or texting a health care provider, lack of experience using a particular modality, or issues connecting to the internet [107]. Past studies on the perceived benefits and limitations of chatbots for advancing sexual and reproductive health care have also reported concerns related to confidentiality and lack of personal communication [50,107]. Data privacy and confidentiality concerns are especially pertinent given the high use of menstrual cycle and fertility-tracking apps among respondents in the sample. A recent assessment of the privacy policies of popular reproductive health apps found that US-based apps received low privacy and security scores due to their use of IP address tracking and sharing of data with third parties for advertising [108].

While the study indicates a high acceptability of digital health for promoting SRH among LBQ+ women of color, respondents still shared concerns about internet access and a lack of digital literacy skills necessary to adopt digital health. These findings align with recent literature that identifies digital literacy and internet access as social determinants of health [107,109,110]. Advancing digital health equity among LBQ+ women of color is critical for the future use of digital health and AI-enabled chatbots to promote SRH among LBQ+ women of color. To achieve digital health equity and promote SRH access among LBQ+ women of color, steps must be taken to address the concerns about using digital health modalities, such as (1) providing easy-to-understand communication and transparency about user data collection, storage, and use; (2) assessing the content, features, and key design considerations for mobile apps or chatbots tailored to LBQ+ women of color; (3) providing tutorials on how to use the modality; (4) introducing structural interventions to address broadband internet access; and (5) screening and intake to identify preferred modalities for facilitating access and uptake of SRH information and services [66]. Taken together, these steps may be integrated into existing digital health equity frameworks to inform how key stakeholders address digital health equity [63,111].

### Strengths and Limitations

The study’s main strengths were the new empirical insights about LBQ+ women of color in the United States, a population significantly underrepresented in research literature. To our knowledge, this is the first study offering empirical insights about the acceptability, influencing factors, and concerns of using digital health, including telemedicine (eg, video calls on a mobile phone), mHealth (mobile apps and SMS text messaging), and AI-enabled chatbots, to promote access to SRH information and services among LBQ+ women of color in the United States. Research suggests that LBQ+ women of color may experience a greater incidence of cervical, breast, and ovarian cancer in the United States due to the underutilization of preventive SRH services such as HPV vaccination and screening measures [17-19,52]. The findings from this study may be used in developing and implementing digital health interventions to reduce these disparities and advance health equity among LBQ+ women of color in the United States [52-54].

The results of this study are not without limitations. First, while the overall sample (n=285) was fairly large, some subgroup categories may have smaller counts, making statistical comparisons unstable. Second, most respondents in the sample were <45 years of age, had health insurance, and had a usual place of care. This limits the generalizability of the findings to older LBQ+ women of color and those with poor access to health care. Third, given that snowball and convenience sampling were used to recruit study participants, the generalizability of the results is limited. Convenience samples are common in empirical research investigating the experiences of sexual minority adults due to difficulty in reaching the population [1]. Due to factors, such as discrimination, bias, and distrust of institutions conducting health-related research, sexual minority adults of color are more hesitant to engage in research than their White and heterosexual counterparts [112,113]. Nationally representative data that include LBQ+ women of color are needed to advance national public health efforts [114]. This study is the first of its kind in this population and seeks to lay the foundation for communities, researchers, practitioners, and stakeholders seeking to advance SRH equity through digital health modalities [63]. Finally, a key limitation of this study was its reliance on primarily online recruitment strategies. Given that this study was conducted during the COVID-19 pandemic, in-person recruitment options were limited. Most of the sample was comprised of individuals residing in the Northeastern (119/285, 41.8%) and Southern (86/285, 30.2%) United States. Respondents living in the Northeastern United States may have greater regional advantages such as better access to the internet. Future research should include offline venue-based sampling methods to ensure a larger sample that is inclusive of LBQ+ women of color who may be impacted by the digital divide (eg, lacking digital literacy skills, having unreliable internet access at home, and having limited access to nonphone devices) [107,109,115].

### Conclusions and Future Directions

The findings showed that LBQ+ women of color had substantial gaps in their awareness and knowledge of SRH services, such as the HPV vaccine and cervical cancer detection. Women in the sample strongly accepted using digital health modalities (ie, video calls, SMS text messaging, and mobile apps) to support access to SRH information and services. However, concerns about digital literacy and technology access barriers, data privacy and confidentiality, and a lack of emotional warmth and relational connection (ie, affective communication) when using digital health modalities were identified.

To advance SRH equity among LBQ+ women of color using digital health, we provide some recommendations. First, culturally tailored digital health tools and interventions that incorporate intersectional considerations (eg, age and income) should be developed, as the findings showed that these sociodemographic factors shape the comfort with particular modalities. Ensure that digital health innovations include inclusive content tailored to the SRH needs of LBQ+ women of color. For example, in our study, there was high usage of mHealth apps for fertility, menstruation, and birth control. However, previous research indicates that LBQ+ women are less likely than their heterosexual peers to receive information and counseling regarding contraception and family planning at the point of care [116]. Second, health literacy (eg, knowledge and awareness) on SRH should be improved among LBQ+ women of color. Targeted messaging that corrects misperceptions and misinformation about the risk for HPV, HIV, and cervical cancer among LBQ+ women of color is essential. Third, digital health solutions must be designed for varying levels of digital literacy and technology access. For example, providing hybrid approaches, such as incorporating SMS text messaging and mobile app use, may ensure reach across income and age among LBQ+ women of color. Finally, efforts should be made to build trust by providing transparency and education regarding health data privacy and confidentiality. We suggest building this trust through community-based collaborative design processes [59,117]. Taken collectively, these recommendations are essential for ensuring that digital health innovations meaningfully advance SRH equity and improve access, knowledge, and preventive care for LBQ+ women of color.

It is important to situate this study’s findings and the above recommendations within the broader context of recent federal policy changes in the United States. This study was conducted before the US Supreme Court overturned Roe v. Wade in the Dobbs v Jackson case on June 24, 2022. This landmark decision, coupled with executive orders targeting lesbian, gay, bisexual, transgender, and queer (LGBTQ) communities, people of color, and women; the mass deletion of government consumer health information websites and data sets; and sweeping cuts to federal agencies that provide millions of Americans with access to technology, the internet, health services, and information, will undoubtedly have a detrimental impact on the SRH outcomes of LBQ+ women of color in the United States [118-122]. For example, LBQ+ women of color in the study expressed concerns about health data privacy and confidentiality when using digital health for sexual and reproductive health care. We hypothesize that concerns about data misuse and the potential for legal prosecution will vary depending on the types of SRH services accessed [123]. Future digital health equity research among LBQ+ women of color should aim to reach a nationally representative sample to assess state-level differences in digital health acceptance and use, with a particular focus on the impact of structural discrimination (eg, abortion laws).

Owing to the mass deletion of critical government consumer health information websites, particularly those that feature the already limited information targeted at LBQ+ women of color, we anticipate that it will become more difficult to find accurate and trustworthy sources of information online [124-126]. Because HPV and cervical cancer awareness and knowledge scores were low among LBQ+ women of color in the sample, efforts to improve information access, awareness, and knowledge, and combat misinformation will become even more critical [95,127,128]. As indicated by the findings from this study, digital health tailored to the SRH information needs of LBQ+ women of color would increase their comfort in using services. Assessing the SRH information needs and sources of LBQ+ women of color is critical for the future development of digital health equity approaches. Understanding the SRH information needs and sources of this community may enhance our understanding of their values and the gaps in their information world that may be addressed to improve their experiences in accessing and using SRH services [111,129].

When respondents were asked about their concerns with using various digital health modalities to receive SRH information and services, they expressed concerns about internet connectivity issues and their inexperience with using digital health modalities. The recent cancellation of the US Digital Equity Act grants program will likely curb efforts to improve broadband internet access and digital literacy skills in communities disproportionately affected by digital inequity [130]. Research indicates that Black and Latino/a/x adults in the United States are less likely to have internet access at home or to own a desktop or laptop computer compared to White adults [115]. Future digital health information aimed at supporting the SRH of LBQ+ women of color must consider providing educational training for the use of modalities, such as AI-enabled chatbots, to improve digital literacy skills.

For advancing SRH equity among LBQ+ women of color using digital health, it will be essential to ensure that they have the necessary resources and digital literacy skills to adopt these innovative approaches [131]. Investigating multilevel determinants of digital health equity, including policy and structural determinants, among LBQ+ women of color will be essential for the development of future digital health interventions aimed at improving SRH outcomes among these women [111,132]. Finally, efforts to address these new challenges to adopting digital health modalities to promote SRH uptake and engagement among LBQ+ women of color in the United States must incorporate community-based research approaches that center the needs, preferences, and cultural norms of LBQ+ women of color [133,134].

## Supplementary material

10.2196/84393 Multimedia Appendix 1 Survey instrument.

10.2196/84393 Multimedia Appendix 2 Multivariable logistic regression models for comfortability with the use of SMS text messaging to communicate with a health care provider to receive support in accessing sexual and reproductive health services.

10.2196/84393 Multimedia Appendix 3 Multivariable logistic regression models for comfortability with the use of video calls to communicate with a health care provider to receive support in accessing sexual and reproductive health services.

10.2196/84393 Checklist 1 CHERRIES checklist.

## Abbreviations

AI: artificial intelligence
DAP: dapivirine
EPV: events per variable
HPV: human papillomavirus
LBQ+: lesbian, bisexual, and queer
LGBTQ: lesbian, gay, bisexual, transgender, and queer
mHealth: mobile health
PrEP: pre-exposure prophylaxis
SRH: sexual and reproductive health
STI: sexually transmitted infection
