# Supplementary material for: Acceptability and Use of Digital Health and Artificial Intelligence–Enabled Chatbots for Sexual and Reproductive Health Among Lesbian, Bisexual, and Queer Women of Color in the United States: Cross-Sectional Survey Study
Source: J Med Internet Res. 2025 Dec 29;27:e84393. doi: 10.2196/84393 (PMC12747503; doi:10.2196/84393)
Supplement: Multimedia Appendix 1 [file jmir-v27-e84393-s001.docx]

**Multimedia Appendix 1: SURVEY**

**Demographic Information**

1. Do you consider yourself Hispanic or Latino?

- Yes
- No

1. Which of the following describes you? *Select all that apply*

- American Indian or Alaskan Native
- Asian
- Black or African American
- Native Hawaiian or Other Pacific Islander
- White
- Prefer Not to Answer

1. What is your age in years? ________________________________________________________________
2. What sex were you assigned at birth, on your original birth certificate?

- Male
- Female
- Intersex

1. What is your current gender identity?

- Cisgender (non-transgender) woman
- Cisgender (non-transgender) man
- Transgender female/trans feminine
- Transgender male/trans masculine
- Nonbinary (e.g., genderqueer, gender non-conforming, agender, gender fluid)
- You don't have an option that describes my current gender identity, please describe: ________________________________________________

1. Do you think of yourself as:

- Lesbian, gay, homosexual
- Straight or heterosexual
- Bisexual
- Pansexual
- Queer
- Questioning, unsure
- Something else, please describe: ___________________________________________________________________________

1. Do you reside in the United States?

- Yes
- No

1. Which state do you reside in?

________________________________________________________________

1. What is the highest level of education that you have completed?

- Less than a high school diploma
- High school diploma or GED
- Some college or Associate’s degree
- Bachelor’s degree  (e.g., BA, BS, AB)
- Master’s degree (e.g., MA, MS, MPH, MPP, MIA, MPA)
- Doctoral degree (e.g., PhD, MD, MD, SD, EdD, DDS)

1. What is your employment status? *Select all that apply*

- Employed full-time
- Employed part-time
- Not currently employed
- Student
- Something else (please specify):

____________________________________

1. Which category best describes your current annual household income (i.e., per year)? Be sure to include income from all sources, such as salary and wages, child support, interest, public assistance, and pensions.

- Less than $10,000
- $10,000 – 24,999
- $25,000 – 49,999
- $50,000 – 100,000
- More than $100,000

1. Thinking about your entire life, have you had a sexual partner who is a cisgender woman, transgender man, or non-binary assigned female at birth person?

- Yes
- No
- Don’t Know

1. Thinking about the last 12 months, have you had a sexual partner who is a cisgender woman, transgender man, or non-binary assigned female at birth person?

- Yes
- No
- Don’t Know

1. Thinking about your entire life, have you had a sexual partner who is a cisgender man, transgender woman, or non-binary assigned male at birth person?

- Yes
- No
- Don’t Know

1. Thinking about the last 12 months, have you had a sexual partner who is a cisgender man, transgender woman, or non-binary assigned male at birth person?

- Yes
- No
- Don’t Know

**Mobile Phone Use**

This section includes questions about ownership and use of a mobile phone.

1. Do you currently own a mobile phone?

- Yes
- No

1. Are you able to send and receive text messages using your mobile phone?

- Yes
- No

1. On average, how often do you use your phone to send text messages?

- Never
- Less than 1 time per week
- 1-6 times per week
- 1-3 times per day
- 4 or more times per day

1. Do you use mobile applications on your mobile phone? (e.g., social media, navigation apps, health apps, etc.)?

- Yes
- No

1. On average, how often do you use your phone to access mobile applications (e.g., social media, health apps, navigation apps, etc.)?

- Never
- Less than 1 time per week
- 1-6 times per week
- 1-3 times per day
- 4 or more times per day

1. Have you used any of the following types of mobile applications on your phone to support your sexual and reproductive health?

|  | Yes | No |
| --- | --- | --- |
| a. period tracking app |  |  |
| b. patient portal, myChart |  |  |
| c. fertility, pregnancy planning app |  |  |
| d. birth control app |  |  |

1. Are you able to complete video calls on your phone (e.g., FaceTime, Google Meet, WhatsApp, etc.)?

- Yes
- No

1. On average, how often do you use your phone to complete video calls?

- Never
- Less than 1 time per week
- 1-6 times per week
- 1-3 times per day
- 4 or more times per day

**Chatbot Use**

*Chatbots, also known as conversational agents, are artificial intelligence-enabled programs designed to imitate human conversations. Chatbots enable verbal and/or text communication with human users and can generate and retrieve information. Chatbots may be programmed to provide information relevant to specific user groups.*

1. Have you ever used a chatbot or conversational agent for any purpose (even if unrelated to sexual and reproductive health)?

- Yes
- No

1. Have you ever sought sexual and reproductive health support from a chatbot or conversational agent?

- Yes
- No

1. In the past 12 months, have you sought sexual and reproductive health support from a chatbot or conversational agent?

- Yes
- No

1. Would you be willing to use a chatbot to assist you with finding sexual and reproductive health information?

- Yes
- No

**Use of Technologies for Sexual and Reproductive Health Care**

Instructions: For the following statements, please read each sentence replacing the blank space with the listed options for each statement (e.g., text messaging, voice call, mobile app, or video call). For example, "I would feel comfortable communicating with a healthcare provider through text messaging to receive support utilizing sexual and reproductive health services.” Then select your response for each statement.

1. I would feel comfortable communicating with a healthcare provider through _________ to receive support utilizing sexual and reproductive health services.

|  | Disagree | Somewhat Disagree | Undecided | Somewhat Agree | Agree |
| --- | --- | --- | --- | --- | --- |
| a. text messaging |  |  |  |  |  |
| b. video call |  |  |  |  |  |
| c. mobile app |  |  |  |  |  |

1. I would feel comfortable using a _________ to assess my risk for cervical cancer.

|  | Disagree | Somewhat Disagree | Undecided | Somewhat Agree | Agree |
| --- | --- | --- | --- | --- | --- |
| a. text messaging |  |  |  |  |  |
| b. chatbot |  |  |  |  |  |
| c. mobile app |  |  |  |  |  |

1. I would feel comfortable using a _________ to assess my risk for acquiring STIs (e.g., HPV, gonorrhea, chlamydia.

|  | Disagree | Somewhat Disagree | Undecided | Somewhat Agree | Agree |
| --- | --- | --- | --- | --- | --- |
| a. text messaging |  |  |  |  |  |
| b. chatbot |  |  |  |  |  |
| c. mobile app |  |  |  |  |  |

1. Would having the option to use a _________ to seek sexual and reproductive health information when you have an urgent health concern increase your comfort seeking sexual and reproductive services?

|  | Yes | No |
| --- | --- | --- |
| a. chatbot |  |  |
| b. mobile app |  |  |
| c. text messaging |  |  |

1. Would having a _________ tailored to the information needs of LBQ+ women of color increase your comfort utilizing sexual and reproductive services?

|  | Yes | No |
| --- | --- | --- |
| a. chatbot |  |  |
| b. mobile app |  |  |

1. Do you have any concerns about using a mobile app to receive sexual and reproductive health information?

- Yes
- No

1. What are your concerns about using a mobile app to receive sexual and reproductive health information?

________________________________________________________________

________________________________________________________________

________________________________________________________________

1. Do you have any concerns about using a chatbot to receive sexual and reproductive health information?

- Yes
- No

1. What are your concerns about using a chatbot to receive sexual and reproductive health information?

________________________________________________________________

________________________________________________________________

________________________________________________________________

1. Do you have any concerns about using a video call to communicate with a healthcare provider about your sexual and reproductive health needs?

- Yes
- No

1. Do you have any concerns about using a mobile app to communicate with a healthcare provider about your sexual and reproductive health needs?

- Yes
- No

1. What are your concerns about using a mobile app to communicate with a healthcare provider about your sexual and reproductive health needs?

________________________________________________________________

________________________________________________________________

________________________________________________________________

1. Do you have any concerns about using text messages to communicate with a healthcare provider about your sexual and reproductive health needs?

- Yes
- No

1. What are your concerns about using text messages to communicate with a healthcare provider about your sexual and reproductive health needs?

________________________________________________________________

________________________________________________________________

**Sexual and Reproductive Health Information**

The following questions will ask about your experiences seeking sexual and reproductive health information.

1. In the past year, have you looked for information on any of the following topics?

|  | Yes | No |
| --- | --- | --- |
| a. STIs (e.g., HPV, chlamydia, gonorrhea) |  |  |
| b. breast cancer |  |  |
| c. cervical cancer |  |  |
| d. menstrual cycle |  |  |
| e. pregnancy prevention (e.g., birth control, abortion) |  |  |
| f. pregnancy planning; childbirth |  |  |
| g. HIV |  |  |
| h. the health of lesbian, bisexual, queer women |  |  |
| i. sexual violence/assault |  |  |

1. In the past year, where have you looked for information about sexual and reproductive health topics? *Select all that apply*

- social media apps (e.g., Facebook, Twitter, Instagram)
- local hospital or healthcare organization
- local hospital or healthcare organization website
- libraries
- government health information websites (e.g., CDC, NIH, WHO)
- consumer health information websites (e.g., WebMD, MayoClinic, HealthFinder)
- LGBTQ+ community organizations
- friends
- blogs
- sexual or romantic partners
- health care provider
- family members
- church or religious institution
- Other (please specify): ________________________________________

1. What is your most preferred source of sexual and reproductive health information?

- social media apps (e.g., Facebook, Twitter, Instagram)
- local hospital or healthcare organization
- local hospital or healthcare organization website
- libraries
- government health information websites (e.g., CDC, NIH, WHO)
- consumer health information websites (e.g., WebMD, MayoClinic, HealthFinder)
- LGBTQ+ community organizations
- friends
- sexual or romantic partners
- health care provider
- family members
- church or religious institution
- Other (please specify): ______________________________

1. If you could ask two questions about your sexual and reproductive health, what would those questions be?

________________________________________________________________

________________________________________________________________

________________________________________________________________

**Awareness and Feelings about HIV Prevention Medication**

The following questions will ask about your awareness and feelings about HIV prevention medication.

1. Have you ever heard of PrEP (pre-exposure prophylaxis)? PrEP is when HIV-negative people take anti-HIV medications (anti-retrovirals like Truvada) BEFORE HAVING SEX to prevent HIV?

- Yes
- No
- Don’t Know

1. Have you ever heard of dapivirine vaginal ring (also referred as the DAP ring)? The dapivirine vaginal ring is a form of pre-exposure prophylaxis. HIV-negative people with a vagina insert the ring which releases daprivine (a topical anti-retroviral) monthly to prevent HIV.

- Yes
- No
- Don’t Know

1. If a pill (drug/medication) that could be taken daily to prevent transmission of HIV from an HIV-positive sex partner to an HIV-negative partner were available, I would take it

- Yes
- No
- Don’t Know

If respondent selected no,

Why would you not take the pill (drug/medication)? *Select all that apply*

- I’m afraid of potential side effects
- I don’t believe it would work
- I don’t like taking pills daily
- I’m not at risk of HIV infection
- I would be afraid that someone would find out that I was taking it
- I would not want to pay for it

1. If a flexible, silicone ring that could be inserted into a vagina and provide sustained release of an anti-HIV drug monthly to prevent the transmission of HIV from an HIV positive sex partner to an HIV negative partner were available I would use it

- Yes
- No
- Don’t Know

If respondent selected no,

Why would you not take the pill (drug/medication)? *Select all that apply*

- I’m afraid of potential side effects
- I don’t believe it would work
- I don’t like having objects inserted in my vagina
- I’m not at risk of HIV infection
- I would be afraid that someone would find out that I was taking it
- I would not want to pay for it

**Awareness and Knowledge about Human Papillomavirus and Cervical Cancer**

The following questions will ask about your awareness and knowledge of human papillomavirus (HPV) and cervical cancer.

1. Have you ever heard of HPV? HPV stands for Human Papillomavirus

- Yes
- No
- Don’t Know

1. Do you think HPV can cause cervical cancer?

- Yes
- No
- Don’t Know

1. Do you think you can get HPV through sexual contact?

- Yes
- No
- Don’t Know

1. Do you think HPV causes AIDS?

- Yes
- No
- Don’t Know

1. Do you think HPV can go away on its own without treatment?

- Yes
- No
- Don’t Know

1. Have you ever heard of the HPV vaccine or shots to prevent cervical cancer?

- Yes
- No
- Don’t Know

1. Have you ever heard of an HPV test?

- Yes
- No
- Don’t Know

1. Have you ever heard of a pap test?

- Yes
- No
- Don’t Know

1. Do you think an HPV test can detect cervical cancer?

- Yes
- No
- Don’t Know

1. Do you think you think you need a pap test in order to receive a cervical cancer diagnosis?

- Yes
- No
- Don’t Know

1. Do you think you only need one dose of the HPV vaccine?

- Yes
- No
- Don’t Know

1. Do you think you can get the HPV vaccine if you’re over the age of 45?

- Yes
- No
- Don’t Know

**Sexual and Reproductive Health Care Access**

This section includes questions about your experiences accessing sexual and reproductive health care.

1. Do you currently have health insurance or health coverage?

- Yes
- No
- Don’t Know

1. What type of health insurance do you have? *Select all that apply*

- A private health insurance plan (such as from an employer or workplace, purchased directly, or through a state or local government or community program)
- Medicaid
- Medicare
- Military health care
- Indian Health Service
- Single-service plan (e.g., dental, vision, prescriptions)
- State-sponsored health plan
- Other government health care (please specify):  __________________________________________________

1. Is there a place that you usually go when you need advice or medical assistance with your sexual and reproductive health needs?

- Yes
- No
- Prefer not to say

1. What kind of place do you go to most often?

- Private doctor’s office
- Community health clinic, community clinic, public health clinic
- Planned Parenthood or family planning clinic
- college or university-based clinic
- hospital emergency room
- urgent care clinic
- Sexually transmitted infection (STI) clinic
- In-store health clinic (e.g., CVS, Walgreens, Target Walmart)
- some other place (please specify): _________________________________________
- doesn't go to one place most often

1. Have you gone to this place in the last 12 months?

- Yes
- No
- Don’t Know

1. What kind of place would you **prefer** to go when you have sexual and reproductive health needs?

- community health clinic, community clinic, public health clinic
- private doctor's office
- Planned Parenthood or family planning
- college or university-based clinic
- hospital emergency room
- urgent care clinic
- Sexually transmitted infection (STI) clinic
- In-store health clinic (e.g., CVS, Walgreens, Target Walmart)
- some other place
- no preference

**Sexual and Reproductive Health Care Use**

This section includes questions about your sexual and reproductive health care experiences. We will first ask about your experience with birth control.

1. Have you ever received a birth control method (e.g., pill, patch, ring, implant) or a prescription for a birth control method from a health care provider?

- Yes
- No
- Don't know

1. Have you received a birth control method (e.g., pill, patch, ring, implant) or a prescription for a birth control method from a health care provider in the last 12 months?

- Yes
- No
- Don't know

1. In your lifetime, have you ever received a Pap test, where a doctor or nurse put an instrument in the vagina and took a sample to check for abnormal cells that could turn into cervical cancer?

- Yes
- No
- Don't know

If respondent selected no,

Why have you not ever had a Pap test? *Select all that apply*

- I don't have health insurance
- I was never offered a Pap test
- I don’t believe it’s necessary if I’m not sexually active with cisgender men
- I was told by a friend or partner it wasn't necessary if I’m not sexually active with cisgender men
- I was told by a health care provider it wasn’t necessary if I’m not sexually active with cisgender men
- I don't know where to get one
- I've had bad experiences getting a Pap test in the past
- Some other reason (please specify): __________________________________________________

1. When was your last Pap test?

- A year ago or less
- More than 1 year ago, but not more than 3 years ago
- More than 3 years ago, but not more than 5 years ago
- Over 5 years ago
- Don't know

1. Have you ever had a Pap test where the results were not normal (e.g., ASC-US, AGC, LSIL, HSIL)?

- Yes
- No
- Don't know

1. In your lifetime, have you ever received an HPV test? An HPV test is sometimes given with the Pap test for cervical cancer screening or during a routine pelvic exam.

- Yes
- No
- Don't know

1. When was your last HPV test?

- A year ago or less
- More than 1 year ago, but not more than 3 years ago
- More than 3 years ago, but not more than 5 years ago
- Over 5 years ago
- Don't know

1. Have you ever tested positive for HPV?

- Yes
- No
- Don't know

HPV is a common sexually transmitted virus that can cause genital warts and cervical and other types of cancer. Vaccines to prevent some HPV infections are available for people 9-45 years of age and are sometimes called the HPV shot, Cervarix, or Gardasil.

1. Have you ever received a shot or dose of the HPV vaccine, also known as the HPV shot, Cervarix, or Gardasil?

- Yes
- No
- Don't know

1. How old were you when you received the HPV vaccine or shot?

- 9-12 years
- 13-17 years
- 18-26 years
- 27-45 years
- Don't know

1. How many shots or doses of the HPV vaccine have you received?

- 1
- 2
- 3
- More than 3
- Don't know

1. How old were you when you received your first dose of the HPV vaccine?

________________________________________________________________

1. Not counting tests you may have had as part of donating blood or blood products, have you ever been tested for HIV?

- Yes
- No
- Don't know

1. At any time in your life, have you ever been told by a doctor or other health care provider that you had a sexually transmitted infection (i.e., STI or STD), like chlamydia, gonorrhea, herpes, trichomoniasis, or syphilis?

- Yes
- No
- Don't know

1. Please identify the STI or STD for which you have received a positive diagnosis *Select all that apply*

- Genital herpes
- trichomoniasis
- gonorrhea
- syphilis
- chlamydia
- HIV

1. Have you ever been pregnant?

- Yes
- No
- Not sure

1. Have you ever given birth?

- Yes
- No

**Healthcare Experiences**

This section includes questions about your experiences obtaining healthcare.

1. In a health care setting (e.g., doctor’s office, hospital, community health clinic), how often do any of the following things happen to you?

|  | Never | Rarely | Sometimes | Most of the time | Always |
| --- | --- | --- | --- | --- | --- |
| You are treated with less courtesy than other people |  |  |  |  |  |
| You are treated with less respect than other people |  |  |  |  |  |
| You receive poorer service than others |  |  |  |  |  |
| A doctor or nurse acts as if he or she thinks you are not smart |  |  |  |  |  |
| A doctor or nurse acts as if he or she is afraid of you |  |  |  |  |  |
| A doctor or nurse acts as if he or she is better than you |  |  |  |  |  |
| You feel like a doctor or nurse is not listening to what you were saying |  |  |  |  |  |

1. What do you think is the main reason for these experiences? *Select all that apply*

- Your gender identity
- Your sexual orientation
- Your race or ethnicity
- Your age
- Your religion
- Your disability
- Your weight
- Your skin color
- Your mental health
- Some other aspect of your physical appearance (please specify): __________________________________________________
- Your education level
- Your income level

1. Have you ever been discriminated against in a health care setting (e.g., hospital, doctor’s office, community health clinic) because of who you are, including but not limited to your ancestry, nationality, religion, gender, sexuality, age, weight, disability, mental health, skin color, education, and/or income?

- Yes
- No
- Not sure

1. Has fear of being discriminated against because of who you are (including but not limited to your ancestry, nationality, religion, gender, sexuality, age, weight, disability, mental health, skin color, education, and/or income) ever caused you to delay obtaining health care?

- Yes
- No
- Not sure

**Thank you for completing the survey and contributing to this important research!**
