# Supplementary material for: Acceptability and Use of Digital Health and Artificial Intelligence–Enabled Chatbots for Sexual and Reproductive Health Among Lesbian, Bisexual, and Queer Women of Color in the United States: Cross-Sectional Survey Study
Source: J Med Internet Res. 2025 Dec 29;27:e84393. doi: 10.2196/84393 (PMC12747503; doi:10.2196/84393)
Supplement: Multimedia Appendix 2 [file jmir-v27-e84393-s002.docx]

Multimedia Appendix 2. Multivariable logistic regression models for comfortability with using SMS text messaging to communicate with a health care provider to receive support in accessing sexual and reproductive health services.

| **Predictor** | **Category** | **Agree** | **Did not agree** | **Adjusted OR (95% CI)** | **p-value** |
| --- | --- | --- | --- | --- | --- |
| **Age** | < 45 (reference) | 71 | 150 | 1.00 | — |
|  | ≥ 45 | 52 | 12 | 1.15 (0.23–5.81) | 0.87 |
| **Income** | ≥ $50,000 (reference) | 56 | 106 | 1.00 | — |
|  | <$ 49,000 | 67 | 56 | 2.10 (0.95–4.67) | 0.068 † |
| **Education** | < Bachelor’s degree (reference) | 80 | 105 | 1.00 | — |
|  | ≥ Bachelor’s degree or higher | 43 | 57 | 1.78 (0.33–9.48) | 0.50 |
| **Usual Source of Care** | Yes (reference) | 75 | 103 | 1.00 | — |
|  | No | 48 | 59 | 1.65 (0.26–10.3) | 0.59 |
| **Insurance** | Insured (reference) | 55 | 158 | 1.00 | — |
|  | Uninsured | 68 | 4 | 0.45 (0.09–2.26) | 0.33 |
| **Region** | Northeast (reference) | 54 | 65 | 1.00 | — |
|  | Midwest | 18 | 25 | 0.68 (0.32–1.45) | 0.32 |
|  | South | 38 | 48 | 0.91 (0.26–3.17) | 0.88 |
|  | West | 13 | 24 | 0.72 (0.14–3.60) | 0.69 |
