# Supplementary material for: Acceptability and Use of Digital Health and Artificial Intelligence–Enabled Chatbots for Sexual and Reproductive Health Among Lesbian, Bisexual, and Queer Women of Color in the United States: Cross-Sectional Survey Study
Source: J Med Internet Res. 2025 Dec 29;27:e84393. doi: 10.2196/84393 (PMC12747503; doi:10.2196/84393)
Supplement: Multimedia Appendix 3 [file jmir-v27-e84393-s003.docx]

Multimedia Appendix 3. Multivariable logistic regression models for comfortability with using video calls to communicate with a health care provider to receive support in accessing sexual and reproductive health services.

| **Predictor** | **Category** | **Agree** | **Did not agree** | **Adjusted OR (95% CI)** | **p-value**^a^ |
| --- | --- | --- | --- | --- | --- |
| **Age** | < 45 (reference) | 179 | 42 | 1.00 | — |
|  | ≥ 45 | 54 | 10 | 1.79 (0.14–22.7) | 0.65 |
| **Income** | ≥ $50,000 (reference) | 133 | 29 | 1.00 | — |
|  | < $49,000 | 100 | 23 | 0.90 (0.26–3.13) | 0.87 |
| **Education** | < Bachelor’s degree (reference) | 133 | 52 | 1.00 | — |
|  | ≥ Bachelor’s degree | 100 | 0 | ~0 (unstable) | 0.99 |
| **Usual Source of Care** | Yes (reference) | 132 | 46 | 1.00 | — |
|  | No | 101 | 6 | ~2.2e6 (unstable) | 0.99 |
| **Insurance** | Insured (reference) | 211 | 2 | 1.00 | — |
|  | Uninsured | 22 | 50 | 0.54 (0.04–6.84) | 0.63 |
| **Region** | Northeast (reference) | 106 | 13 | 1.00 | — |
|  | Midwest | 32 | 11 | 0.37 (0.14–0.98) | *0.045* |
|  | South | 80 | 6 | 1.71 (0.18–16.6) | 0.64 |
|  | West | 15 | 22 | 0.09 (0.01–1.49) | 0.094 |

^a^ Italicized *P* value denotes statistical significance
