# Supplementary material for: Acceptability and Use of Digital Health and Artificial Intelligence–Enabled Chatbots for Sexual and Reproductive Health Among Lesbian, Bisexual, and Queer Women of Color in the United States: Cross-Sectional Survey Study
Source: J Med Internet Res. 2025 Dec 29;27:e84393. doi: 10.2196/84393 (PMC12747503; doi:10.2196/84393)
Supplement: Checklist 1 [file jmir-v27-e84393-s004.docx]

CHERRIES (Checklist for Reporting Results of Internet-Based e-Surveys) checklist.

| ***Item Category*** | ***Checklist Item*** | ***Explanation*** |
| --- | --- | --- |
| **Design** |  |  |
|  | Describe survey design | A convenience sample. |
| **IRB (Institutional Review Board) approval and informed consent process** | IRB approval | IRB approval was obtained from Rutgers University. This study was determined to pose minimal risk to participants. (IRB #2020002014) |
|  | Informed consent | Megan Threats was the principal investigator (PI). Deidentified survey data was stored on password protected cloud servers and access was restricted to the PI.  Interested respondents were informed of the purpose of the survey, estimated length of time of the survey, anonymity, confidentiality, benefits, risks, and voluntarily filled out the electronic informed consent form before beginning the main survey instrument. |
|  | Data protection | The survey was anonymous. IP addresses were not collected. The PI extracted an excel file from Qualtrics with respondent data that was downloaded to a university-approved and secured cloud server. The drive was password-protected and accessible only to the PI. Only the PI was able to access, view and analyze the survey data. |
| **Development and pre-testing** | Development and testing | The survey was tested with four members of the target population who completed the survey. These individuals did not participate in the study. The survey items were adapted from previously validated scales and/or published survey instruments. |
| **Recruitment process and description of the sample having access to the questionnaire** | Open survey versus closed survey | Open survey, but the link was anonymous and only shared with individuals who received a copy of the link. |
|  | Contact mode | Initial contact with the potential respondents was done via postings on social media sites and through email list servs. |
|  | Advertising the survey | Flyers advertising the survey were posted on social media sites including Twitter, Instagram, and Facebook. Flyers advertising the survey were also shared via the email list servs of community-based organizations and university list servs. The survey flyer included information about the survey, its timing, and how to access it. |
| **Survey administration** | Web/E-mail | The survey was sent out via email and responses were collected via Qualtrics. |
|  | Context | N/A |
|  | Mandatory/voluntary | Voluntary |
|  | Incentives | Respondents who completed the survey had the option to enter a drawing to receive one of seventy-five available $35 virtual gift cards. The drawing was based entirely on chance and each participant had equal odds of winning. |
|  | Time/Date | Data was collected from November 2020 up to March 2021. |
|  | Randomization of items or questionnaires | Question order was not randomized. |
|  | Adaptive questioning | Yes |
|  | Number of Items | The number of survey items per page ranged from two to three. |
|  | Number of screens (pages) | N/A |
|  | Completeness check | A completeness check was implemented by Qualtrics. Respondents were prompted to check non-applicable or answer the question. |
|  | Review step | Respondents were not able to go ‘back’ in the survey to review and change their answers. |
| **Response rates** |  |  |
|  | Unique site visitor | 456 |
|  | View rate (Ratio of unique survey visitors/unique site visitors) | N/A |
|  | Participation rate (Ratio of unique visitors who agreed to participate/unique first survey page visitors) | 350/456 (76.8%) |
|  | Completion rate (Ratio of users who finished the survey/users who agreed to participate) | 285/350 (81.4%) |
| **Preventing multiple entries from the same individual** | Cookies used | Cookies/embedded data were used to assign a unique user identifier to each client computer. The cookies were set and read when they accessed the survey. It expired when they completed the survey or after 6 months. Duplicate entries from the same user were prevented using the following Qualtrics features “bot detection”, “RelevantID”, and “In Survey: Prevent multiple submissions.” |
|  | IP check | There was no IP check done to prevent multiple or duplicate entries from the same user. No IP addresses were collected to anonymize responses. The following Qualtrics features were used to minimize entries from the same individual: “bot detection”, “RelevantID”, and “In Survey: Prevent multiple submissions.” |
|  | Log file analysis | N/A |
|  | Registration | N/A |
| **Analysis** | Handling of incomplete questionnaires | Only completed surveys were included in the data analysis. |
|  | Questionnaires submitted with an atypical timestamp | N/A |
|  | Statistical correction | The dependent variable in this study was non-normal distributed and therefor binary logistic regression was used. |
